# Cellular infiltration in an injectable sulfated cellulose nanocrystal hydrogel and efficient angiogenesis by VEGF loading

**DOI:** 10.1186/s40824-023-00373-y

**Published:** 2023-04-10

**Authors:** Kiyoon Min, Giyoong Tae

**Affiliations:** School of Materials Science and Engineering, Gwangju Institute of Science and Technology, 123 Cheomdan-Gwagiro, Buk-Gu, Gwangju, 61005 Republic of Korea

**Keywords:** Cellulose nanocrystal (CNC), Injectable hydrogel, Vascular endothelial growth factor, Cellular infiltration, Angiogenesis

## Abstract

**Background:**

Cellular infiltration and angiogenesis into implanted biomaterial scaffolds are crucial for successful host tissue integration and tissue regeneration. Cellulose nanocrystal (CNC) is a nano-sized cellulose derivative, which can form an injectable physical gel with salts. Sulfate groups of sulfated CNC (CNC-S) can act as a binding domain to various growth factors and cytokines with a heparin-binding domain for sustained release of them. Vascular endothelial growth factor (VEGF) can promote the proliferation of endothelial cells and angiogenesis. In this study, VEGF-loaded CNC-S hydrogel was evaluated as an injectable scaffold that can induce cellular infiltration and angiogenesis.

**Methods:**

CNC-S was hydrolyzed to get desulfated CNC (CNC-DS), which was used as a negative control group against CNC-S. Both CNC-S and CNC-DS hydrogels were prepared and compared in terms of biocompatibility and VEGF release. The hydrogels with or without VEGF loading were subcutaneously injected into mice to evaluate the biocompatibility, cellular infiltration, and angiogenesis induction of the hydrogels.

**Results:**

Both hydrogels possessed similar stability and shear-thinning behavior to be applicable as injectable hydrogels. However, CNC-S hydrogel showed sustained release (until 8 weeks) of VEGF whereas CNC-DS showed a very fast release of VEGF with a large burst. Subcutaneously injected CNC-S hydrogel showed much enhanced cellular infiltration as well as better biocompatibility with milder foreign body response than CNC-DS hydrogel. Furthermore, VEGF-loaded CNC-S hydrogel induced significant angiogenesis inside the hydrogel whereas VEGF-loaded CNC-DS did not.

**Conclusion:**

CNC-S possesses good properties as a biomaterial including injectability, biocompatibility, and allowing cellular infiltration and sustained release of growth factors. VEGF-loaded CNC-S hydrogel exhibited efficient angiogenesis inside the hydrogel. The sulfate group of CNC-S was a key for good biocompatibility and the biological activities of VEGF-loaded CNC hydrogel.

**Supplementary Information:**

The online version contains supplementary material available at 10.1186/s40824-023-00373-y.

## Background

Angiogenesis is a prerequisite for biomaterials used in tissue regeneration since the blood vessels and their networks are essential for delivering oxygen and nutrients, removing wastes, and making connections to the surrounding tissues [1, 2]. To these necessities, many biomaterial scaffolds have been proposed to provide the sites of cellular infiltration on the implanted lesions [3, 4]. Physical hydrogels generally have the intrinsic potential for injectability and cell infiltration due to their reversible and less stable cross-linking [5, 6], whereas chemically cross-linked (covalently cross-linked) hydrogel systems require special chemical functionality for injectability and additional modification to provide degradation or large porosity for cell ingrowth [7–9]. The injectability of physical hydrogels enables the delivery of a large volume through minimally invasive administration and their structural recovery after stress conditions during injection [5, 10]. Also, cellular infiltration into acellular biological matrix can accelerate tissue regeneration by promoting structural remodeling and host tissue integration [11].

Furthermore, diverse growth factors/cytokines-loaded biomaterials have been studied to induce angiogenesis inside scaffolds. For example, vascular endothelial growth factor (VEGF)-loaded, integrin-modified polyethylene glycol (PEG) hydrogels showed vascularization inside the implanted hydrogels and bone repairing a bone defect model [12], and basic fibroblast growth factor (bFGF)-loaded hyaluronic acid hydrogel allowed cellular infiltration and ingrowth of host tissues by subcutaneous implantation into rats [13]. Especially, VEGF has been known as a key factor to promote the proliferation of the endothelial cell, angiogenesis, recovery, and regeneration of tissues [14, 15]. However, due to its short half-life in vivo, the proper delivery strategies for sustained release of VEGF as a bioactive state are necessary.

Many growth factors, such as VEGF, bFGF, epidermal growth factor (EGF), hepatocyte growth factor (HGF), and bone morphogenetic protein-2 (BMP-2) have a heparin-binding domain, so heparin-functionalization has been widely used for sustained release of growth factors as bioactive states [16–20]. Since a high density of sulfate group in heparin is a key characteristic of the biological activities of heparin including the binding affinity to various growth factors, introducing sulfate groups has been applied as a useful strategy to make heparin-mimicking modification [21, 22].

Cellulose is an environmentally degradable and most abundant polysaccharide on earth, so it has been in the spotlight as an eco-friendly natural material [23–25]. Cellulose nanocrystal (CNC), one of the processible cellulose derivatives, is a rod-like nanoparticle made from cellulose through acid hydrolysis [26]. CNC has several advantages as biomaterials due to its nano-size, good mechanical properties, high surface area, and ease of chemical modification [27]. CNC could also have sulfate groups during the preparation process from cellulose. Thus, it is expected that sulfated CNC (CNC-S) can load growth factors efficiently and provide sustained release of them. CNC itself can form a hydrogel state in the presence of salt by the rearrangement of the physical assembly of CNC [28], and the shear-thinning behavior of the physical assembly of CNC enabled its use as an injectable gel [29].

Accordingly, cytotoxicity and biocompatibility of CNC-containing materials have been studied to evaluate their potential for biomedical applications, and the results showed acceptable biocompatibility in general. For example, collagen/CNC composite scaffolds showed good biocompatibility both in vitro and in vivo [30]. The Cranston group reported no significant cytotoxicity by CNC conjugation to carboxymethyl cellulose and dextran-based hydrogel [31]. They also studied tissue response and biodistribution of injectable PEG-based hydrogels containing CNC at various CNC concentrations, showing moderate acute inflammation even for the highest concentration (4.95 wt%) of CNC while mild or no inflammation for the lower concentration groups in vivo [26]. However, in vivo evaluation of the biocompatibility of CNC itself and CNC-based hydrogel, not as an additive to other materials, has been rarely reported; the biocompatibility of a low concentration (0.5%) CNC suspension, not as a hydrogel, was compared with cellulose nanofiber [32]. Also, the biocompatibility and biodistribution of amine-functionalized CNC as a nanoparticle state after labeling with radioactive metal and fluorescent dye upon intravenous injection into mice were analyzed [33].

**Scheme 1:**
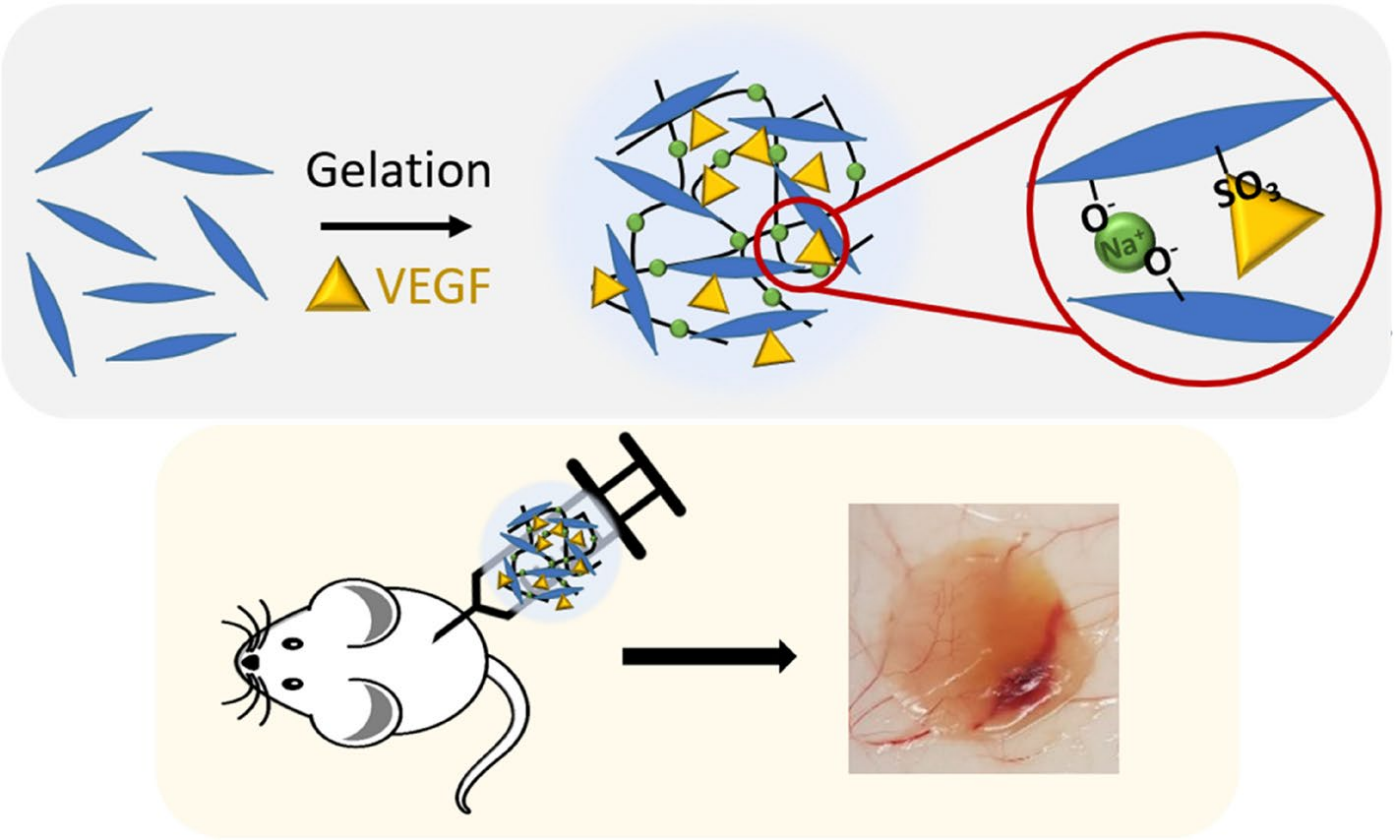
VEGF loaded into injectable CNC-S hydrogels were subcutaneously injected into mice to induce cellular infiltration and angiogenesis

In this study, by utilizing the injectable and physical gel state of CNC-S in physiological conditions, we evaluated the release of VEGF from CNC-S gel in vitro, cellular infiltration, and the induction of angiogenesis inside the VEGF-loaded CNC-S gel upon subcutaneous injection in vivo. By comparing with desulfated CNC (CNC-DS) gel as a negative control group, the role of the sulfate group in CNC-S was elucidated (Scheme 1). This study would provide the high potential of CNC-S gel as a biologically active hydrogel for tissue regeneration as well as for the delivery of growth factors and cytokines.

## Methods

### Materials

Sulfated cellulose nanocrystal (CNC-S) extracted from wood pulp (MW 14,700–27,850, sulfur content 0.86–0.89%, sulfate content 246–261 mmol/kg and 0.60–0.65 μmol/m^2^, particle diameter 2.3–4.5 nm, particle length 44–108 nm) was kindly donated from CelluForce (Quebec, Canada). Sodium chloride, potassium chloride, sodium phosphate, and potassium phosphate for phosphate-buffered saline (PBS) were purchased from Sigma-Aldrich (St. Louis, MO, USA). Human recombinant vascular endothelial growth factor (VEGF) 165 and VEGF ELISA kit were bought from R&D Systems (Minneapolis, MN, USA). Human umbilical vein endothelial cells and endothelial basal media were bought from Lonza (Basel, Switzerland), and Cell Counting Kit-8 (CCK-8) was purchased from Dojindo Molecular Technologies, Inc. (Rockville, MD, USA). Hematoxylin and eosin Y were bought from BBC Biochemical (Mount Vernon, WA, USA), and Masson’s trichrome staining kit was obtained from Diagnostic Biosystems (Pleasanton, CA, USA). Xylene and ethanol for the dehydration of tissues were bought from Duksan (Gyeonggi-do, Korea). Anti-CD68, anti-CD31, anti-αSMA, and anti-rabbit Alexa 594 antibodies were purchased from Abcam (Cambridge, UK), and anti-rabbit Alexa 594 for the secondary antibody of immunofluorescence staining, DAPI, and mounting solution were bought from Invitrogen (Carlsbad, CA, USA).

### Desulfation of sulfated cellulose nanocrystal

Desulfation of CNC-S was processed following the modified method of the previous report [34]. 500 mg of CNC-S was dissolved in 50 mL of deionized water (DIW) (1 wt%) and preheated to 80 °C. 2.5 mL of 5 N hydrochloric acid (HCl) was added to the CNC-S solution to set the final concentration of HCl as 0.25 N. The solution was stirred and heated at 80 °C in a water bath overnight. The solution was cooled down to room temperature and dialyzed against DIW to remove the remaining ions for 2 days. The final product was freeze-dried and stored at -20 °C for further experiments. Desulfation of CNC was analyzed by Fourier-transform infrared spectrometer (FT-IR, Vertex 70v, Bruker, Billerica, MA, USA) and X-ray photoelectron spectroscopy (XPS, NEXSA, Thermo Fisher Scientific, Waltham, MA, USA). The sulfur contents of CNC-S and CNC-DS were analyzed by an elemental analyzer (EA, UNICUBE, Elementar, Langenselbold, Germany), and the zeta potentials of CNC-S and CNC-DS were measured by dynamic laser scattering (ELSZ-2000, Otsuka Electronics, Osaka, Japan).

The morphologies of cellulose nanocrystals were observed using a transmission electron microscope (TEM, Tecnai G2 F30 S-Twin, FEI, Hillsboro, OR, USA), and the particle sizes were measured from images by ImageJ software (*n* = 20). The size distribution of CNC-S and CNC-DS dispersed in DIW with 0.05% Tween 80 before gelation was measured using dynamic light scattering.

### Preparation of cellulose nanocrystal (CNC) hydrogel

CNC-S and CNC-DS stocks were dissolved in 5 wt% in DIW, followed by 10 min ultrasonication (Vibra cell, SONICS & Materials Inc., Newtown, CT, USA) with 10 s on/10 s off pulses for homogeneous dispersion. An injectable hydrogel state was prepared by mixing 80 μL of 5 wt% CNC-S or CNC-DS with 10 μL of 10 X PBS and 10 μL of DIW to get 100 μL of 4 wt% CNC in 0.1 M (100 mM) PBS. The injectable gel was loaded into a syringe and injected through a 22 G needle.

### Characterizations of CNC hydrogels

The microstructures of CNC hydrogels were observed using a scanning electron microscope (SEM, JSM-7500F, Jeol, Tokyo, Japan). The hydrogels were washed with DIW to remove salts and freeze-dried for SEM analysis.

The rheological properties of CNC-S and CNC-DS hydrogels were analyzed by a rheometer (Kinexus, Malvern Instrument, UK) with a 15 mm diameter of plate geometry sample holder and 0.5 mm of gap thickness. The frequency sweep test was done from 0.1 to 100 rad/s with 0.1% of strain at 37 °C. Also, to demonstrate their injectability and shear thinning behavior, viscosity was measured both by varying the frequency from 0.1 to 100 and the shear rate from 0.01 to 100 with the same geometry and cone-shaped geometry, respectively. The same analyses were conducted at various NaCl concentrations (0, 5, 10, 25, 50, 100 mM) of PBS.

### In vitro stability

The in vitro stability of CNC-S and CNC-DS hydrogels was observed by making the hydrogels (100 μL) at the bottom of 1.5 mL Eppendorf tubes, adding excess PBS, and measuring their remaining heights over time while applying gentle shaking (100 rpm) in a 37 °C shaker.

### In vitro loading and release of VEGF from CNC hydrogel

VEGF was loaded into CNC hydrogels (CNC-S and CNC-DS) by simple addition before salt-induced gelation. To prepare CNC hydrogel containing VEGF (1 μg/100 μl), 100 X excess volume of PBS with 0.05% BSA and 0.05% sodium azide was added as a release media for loaded VEGF. The setup was put in a 37 °C shaker with gentle shaking (100 rpm), and the whole release media was replaced with the fresh one regularly. The released amount of VEGF was measured from the initial burst (10 min) to 60 days (2 months) by VEGF ELISA.

To analyze the bioactivity of released VEGF from CNC-S or CNC-DS hydrogel, released VEGF in cell culture media for 24 h (after an initial 1 h) was collected and the concentration of VEGF was measured by VEGF ELISA. Then, VEGF released from the gel or pristine VEGF (5 ng/ml) was added to 7,000 cells of HUVEC (passage 5) on a 96-well plate, and the cells were cultured using the endothelial basal media from Lonza with the addition of 2% FBS but without the addition of supplementary growth media. After 48 h, the metabolic activities of HUVECs with or without exogenously added VEGF were measured using a CCK-8 assay.

### In vivobiocompatibility evaluation

In vivo experiments using balb/c mice (male, 8 week-old) were proceeded following the guideline of the Animal Care and Use Committee of Gwangju Institute of Science and Technology (GIST-2020–114). 8 week-old balb/c mice were obtained from G-bio (Gyeonggi-do, Korea). Acclimatization was provided for one week to the mice with no restriction on food, water, or behavior and a 12-h/12-h day-night cycle. To evaluate the biocompatibility of the CNC hydrogels, 100 μL hydrogels (subcutaneous injection of that volume of hydrogel to mice was acceptable according to the guideline for animal experiments) were subcutaneously injected into each left and right flank of randomized hair-removed mice (two gels per mouse, two mice per group and each time point). After 2 and 4 weeks, the mice were sacrificed and the skins with hydrogels (each *n* = 4) were extracted and fixed with 4% formaldehyde. The skins and gels were dehydrated by submerging them in 30% sucrose in PBS and embedded in an optimal cutting temperature compound (OCT). The tissue slices were sectioned in 10 $\mathrm{\mu m}$ thickness by a cryotome (Leica, Wetzlar, Germany). The tissue slides were stained for histological analysis. Hematoxylin and eosin Y (H&E) staining was proceeded to observe the structure of tissues and hydrogels, and Masson’s trichrome staining was carried out to see collagen layer deposition due to foreign body reaction. For immunofluorescence staining, the slides were permeabilized using 0.25% of Triton X-100, followed by blocking steps with 1% of BSA in PBS. Anti-CD68 antibody for primary antibody and secondary antibody (anti-rabbit Alexa 594) were treated with 200 times dilution for macrophage staining. Then, DAPI was treated for 5 min and the slides were mounted with a water-soluble mounting solution. The stained tissues were observed by a research slide scanner (VS200, Olympus, Tokyo, Japan) and fluorescence microscope (TE2000-U, Nikon Co., Tokyo, Japan), and the staining positive areas were measured using Image J software.

### In vivo bioactivity of VEGF-loaded CNF hydrogels

One μg VEGF-loaded 100 μL CNC hydrogels were subcutaneously injected into each left and right flank of randomized hair-removed mice to demonstrate the in vivo bioactivity of loaded VEGF (two gels per mouse, two mice per group). After 2 weeks, the skins with the CNC hydrogels (*n* = 4) were excised and processed in the same way as the biocompatibility experiment for the immunostaining. The primary antibody for the cluster of differentiation 31 (CD31) and alpha-smooth muscle actin (αSMA) was diluted 50 times and 100 times, respectively, and the secondary antibody (anti-rabbit Alexa 594) was diluted 200 times for fluorescence tag. The stained tissues were imaged by fluorescence microscopy and analyzed using ImageJ software.

### Statistical analysis

Statistical analyses of the data were done by student’s t-test using Microsoft Excel. *p*-values under 0.05 were regarded as significant differences. No criteria were set to exclude the animals and data points during analysis. (#:* p* > 0.05 no significance difference, *: *p* < 0.05, **: *p* < 0.01, ***: *p* < 0.001).

## Results

### Preparation and characterizations of injectable CNC hydrogels

**Fig. 1:**
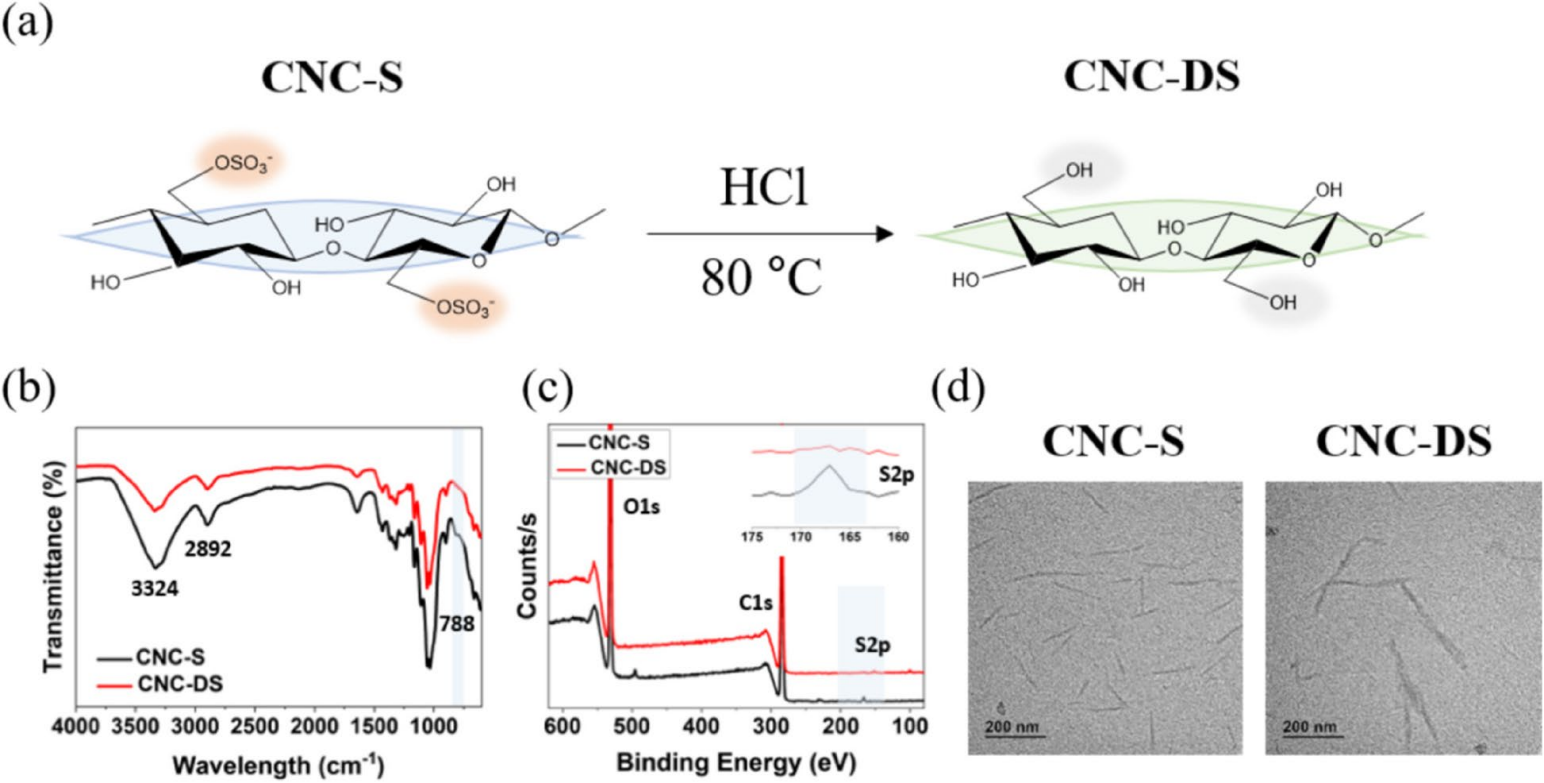
**a** Desulfation process and chemical unit structures of CNC-S and CNC-DS. **b** FT-IR and (**c**) XPS spectra of CNC-S and CNC-DS. **d** The morphologies of CNC-S and CNC-DS particles observed by TEM

CNC-S has sulfate groups obtained from the H_2_SO_4_-hydrolysis process to make nanocrystals (Fig. 1(a)) [34]. To evaluate the role of sulfate groups of CNC as a VEGF carrier, desulfated CNC (CNC-DS) was prepared by the HCl-acid hydrolysis method as a control group for CNC-S. Desulfation of CNC-S was verified by FT-IR and XPS, and their spectra showed the changes in chemical bonds.

FT-IR spectra shown in Fig. 1(b) displayed broad bands at 3324 cm^−1^ and 2893 cm^−1^ of stretching vibrations of OH and CH, respectively, originating from the CNC backbone and hydroxyl groups in both CNC-S and CNC-DS. The peak at 788 cm^−1^ from CNC-S disappeared after the desulfation process, which was attributed to the C-O-S vibration of the sulfate group [35]. Furthermore, the XPS spectra in Fig. 1(c) also proved the apparent desulfation of CNC-S. Both spectra of CNC-S and CNC-DS showed clear O1s and C1s peaks. On the other hand, the peak for S2p was dramatically reduced in CNC-DS. The peak at 168 eV represented the sulfo group (-SO_3_-) [36]. The sulfur content of the CNC-S was 0.8 $\pm$ 0.2% by elemental analysis, but it became negligible after desulfation for CNC-DS, demonstrating the successful desulfation of CNC-S. The zeta potential of CNC-DS was also increased to -25 $\pm$ 1 mV compared to -38 $\pm$ 3 mV of the initial CNC-S due to the substitution of the sulfate group to the hydroxyl group (Table 1). The morphology of each CNC-S and CNC-DS, observed by TEM, showed rod-like shapes and 121 ± 36 nm and 140 ± 31 nm respectively in length, which has no statistical difference between the two groups (Fig. 1(d)). Similar size distributions of CNC-S and CNC-DS in DIW were observed by dynamic light scattering (Fig. S1), also confirming no significant change in the morphology after desulfation. A small size peak (~ 10 nm) seemed to result from the added surfactant (0.05% tween 80) to disrupt aggregation among CNCs [37].

**Table 1:** Sulfur content and zeta potential of CNC-S and CNC-DS

	**Sulfur content (%)**	**Zeta potential (mV)**
CNC-S	0.8 ± 0.2	-38 ± 3
CNC-DS	0.1 ± 0.1^a^	-25 ± 1

^a^This value was under the detection limit of the EA instrument

**Fig. 2:**
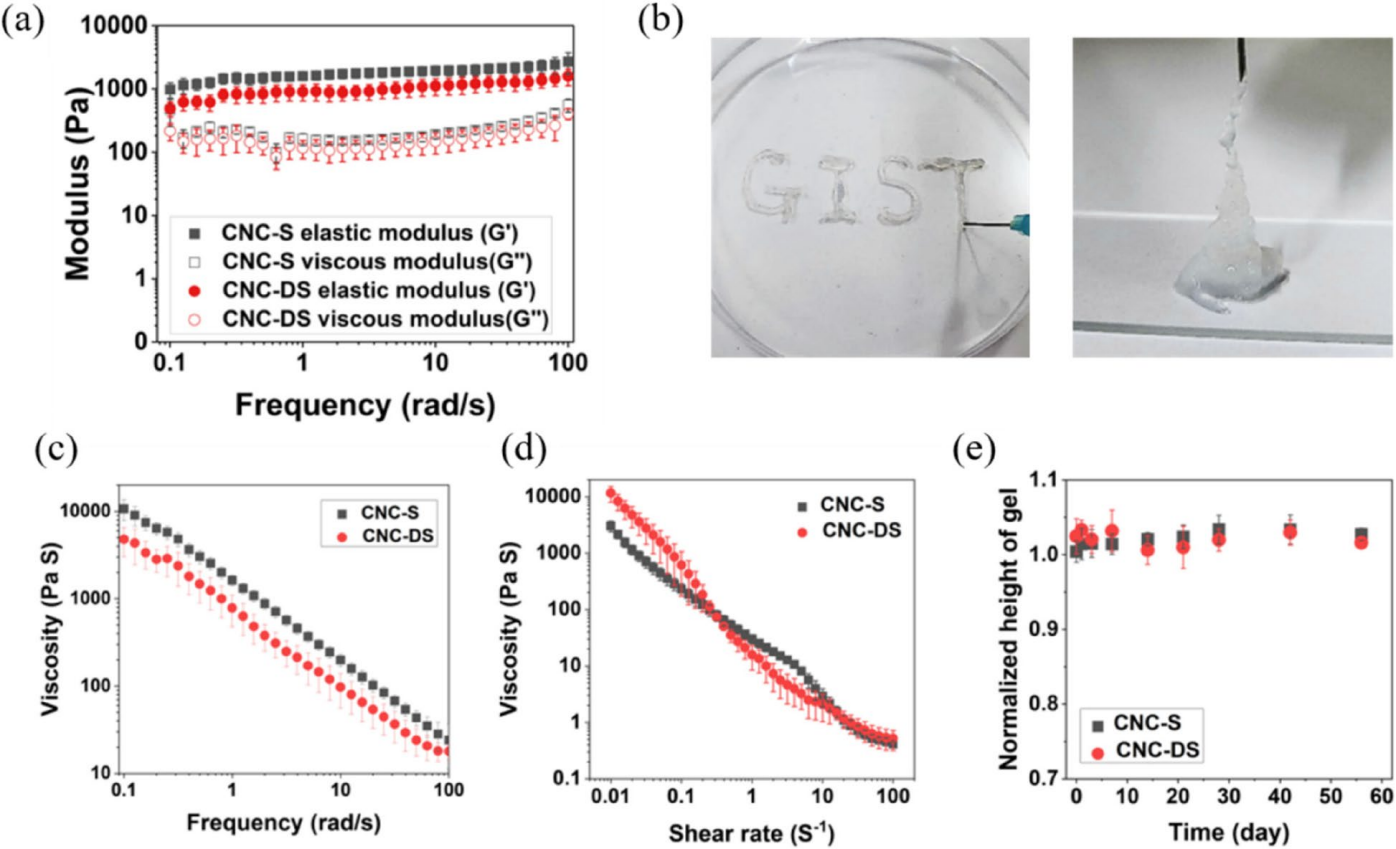
The rheological properties and in vitro stability of injectable CNC hydrogels. **a** The frequency sweep and (**b**, **c**) shear-thinning behaviors of CNC hydrogels. **d** In vitro stability of CNC injectable hydrogels under physiological conditions

The microstructures of freeze-dried CNC hydrogels were observed by SEM (Fig. 1 (b) and (c)). Both CNC-S and CNC-DS showed around 10 μm-sized, connected pores with no distinct difference between them. The rheological properties of CNC hydrogels (4 wt %) were characterized. In the frequency sweep test (Fig. 2(a)), the frequency-independent elastic moduli (G’) that were higher than viscous moduli (G”) were observed for both CNC-S and CNC-DS, supporting the formation of a stable gel state. Both CNC-S and CNC-DS showed G’ of ~ 1 kPa, comparable to that of the brain or soft tissues [38]. Complex viscosity *vs.* frequency and viscosity *vs.* shear rate were measured to demonstrate the injectability of hydrogels (Fig. 2(b) and (c)). Both CNC-S and CNC-DS hydrogels showed shear-thinning behavior, confirming the injectability of CNC hydrogels.

The rheological characteristics of CNC-hydrogels were additionally analyzed with various concentrations of salts in PBS to induce gelation (Fig. S3 and Fig. S4). Since CNC-DS can associate with each other much better than CNC-S via hydrogen bonding and hydrophobic association, the gelation of CNC-S showed much stronger dependence on salt concentration than CNC-DS, and CNC-DS showed a viscous and physical gel state even without salt [39]. However, the dependency of sodium ion concentration was saturated when the ionic strength of the solution was sufficiently high (from 50 mM in both cases), so the rheological responses of both hydrogels were the same at physiological conditions.

In addition, in vitro stability of CNC-S and CNC-DS in PBS with gentle shaking was observed for 8 weeks (Fig. 2(d)). However, no sign of degradation or dissolution of the hydrogels was observed for both CNC-S and CNC-DS, showing the high stability of CNC hydrogels in physiological conditions. Consequently, CNC-S and CNC-DS did not show a noticeable difference in the in vitro physical characteristics, such as microstructures, rheological characteristics, and stability.

### In vitrorelease and bioactivity of VEGF from CNC hydrogel

**Fig. 3:**
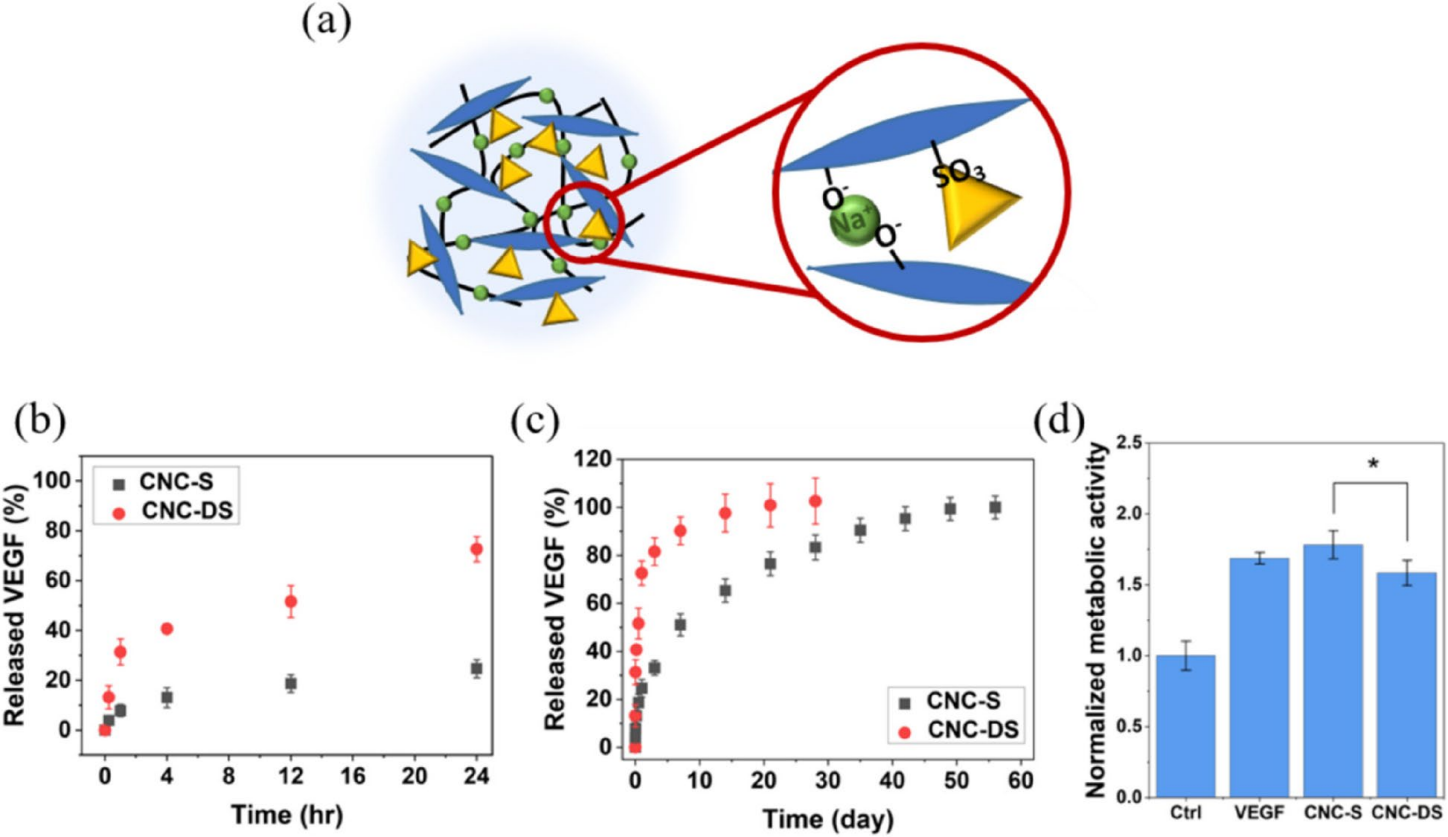
**a** The interaction of CNC, salt, and VEGF in the CNC-S hydrogel (blue: CNC, green circle: salt, and yellow triangle: VEGF). The release profiles of VEGF from CNC hydrogels and the bioactivity of released VEGF. The release profile of VEGF (**b**) for initial 24 h and (**c**) for 8 wk. **d** The bioactivity of released VEGF measured by HUVEC proliferation. *: *p* < 0.05)

VEGF that can induce angiogenesis was loaded into CNC hydrogels by simply adding and mixing (Fig. 3(a)). The amounts of released VEGF at each time point were measured by ELISA. A very fast release of VEGF was observed from CNC-DS with no sulfate group. In contrast, CNC-S revealed a much more sustained release of loaded VEGF compared to CNC-DS, as shown in Fig. 3(b) and (c). The released amount of VEGF from CNC-DS reached almost 75% of the loading amount in 24 h, whereas CNC-S released only ~ 25% of the loaded VEGF at the same time, and it took a month to get ~ 80% release, and about 2 months for total release.

The bioactivity of released VEGF from CNC hydrogels was investigated by the effect of VEGF on HUVEC proliferation in Fig. 3(d). Compared to the control (without VEGF), VEGF released from CNC-S and CNC-DS increased the metabolic activity of HUVEC. Although released VEGF from both cases showed the bioactivity to enhance the proliferation of HUVEC, released VEGF from CNC-S showed slightly higher metabolic activity (~ 1.8 times increase compared to the control) than that from CNC-DS (~ 1.6 times increase compared to the control).

### In vivobiocompatibility of injected CNC hydrogel

**Fig. 4:**
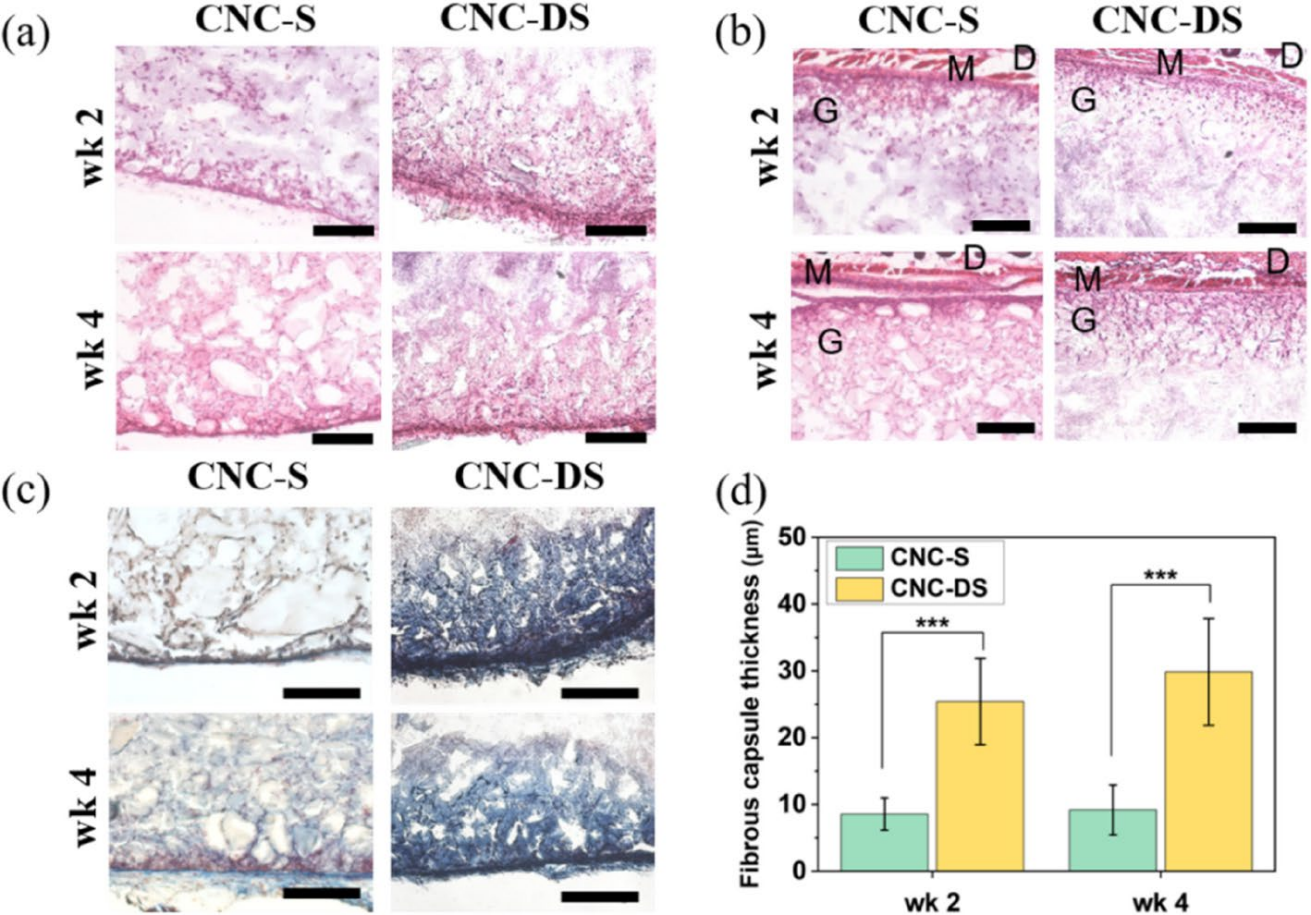
**a** Histological analysis of subcutaneously injected CNC hydrogels. H&E staining images of CNC hydrogels (**a**) and interface between muscle layer (**b**) and Masson’s trichrome staining images of CNC hydrogels (**c**) at wk 2 and wk 4. (Scale bar = 200 μm) (**d**) The thickness of fibrous capsules formed for CNC hydrogels (*n* = 10). (D: dermis, M: muscle, G: gel) (***: *p* < 0.001)

The CNC hydrogels were subcutaneously injected into mice using a 22 G needle without physical surgery. Injected hydrogels were excised with skins after 2 and 4 weeks and the biocompatibility of the hydrogels was analyzed through histological analysis. H&E staining (Fig. 4(a)) showed the infiltrated cells (purple dots) inside the hydrogels from the edge parts of the hydrogels for both CNC-S and CNC-DS hydrogels at week 2 and 4. Also, CNC hydrogels seemed to show good adhesion with surrounding tissues (Fig. 4(b)); both CNC hydrogels were well-adhered under muscle layers. The hydrogels were also stained with Masson’s trichrome staining to visualize the fibrous capsule formation induced by the foreign body reaction. As shown in Fig. 4(c) and (d), the fibrous capsule formation at the edges of the hydrogels was observed in both cases. For CNC-S hydrogels, the thickness of the fibrous capsules was 8.5 $\pm$ 2.4 μm and 9.2 $\pm$ 3.7 μm at week 2 and 4, respectively. On the other hand, CNS-DS hydrogels showed 28.2 $\pm$ 5.5 μm and 24.0 $\pm$ 4.9 μm of fibrous capsule thickness at week 2 and 4, showing ~ 3 times thicker capsule formation than CNC-S. Also, the inside part of the gels near the edges of CNC-DS was stained blue, showing collagen deposition.

**Fig. 5:**
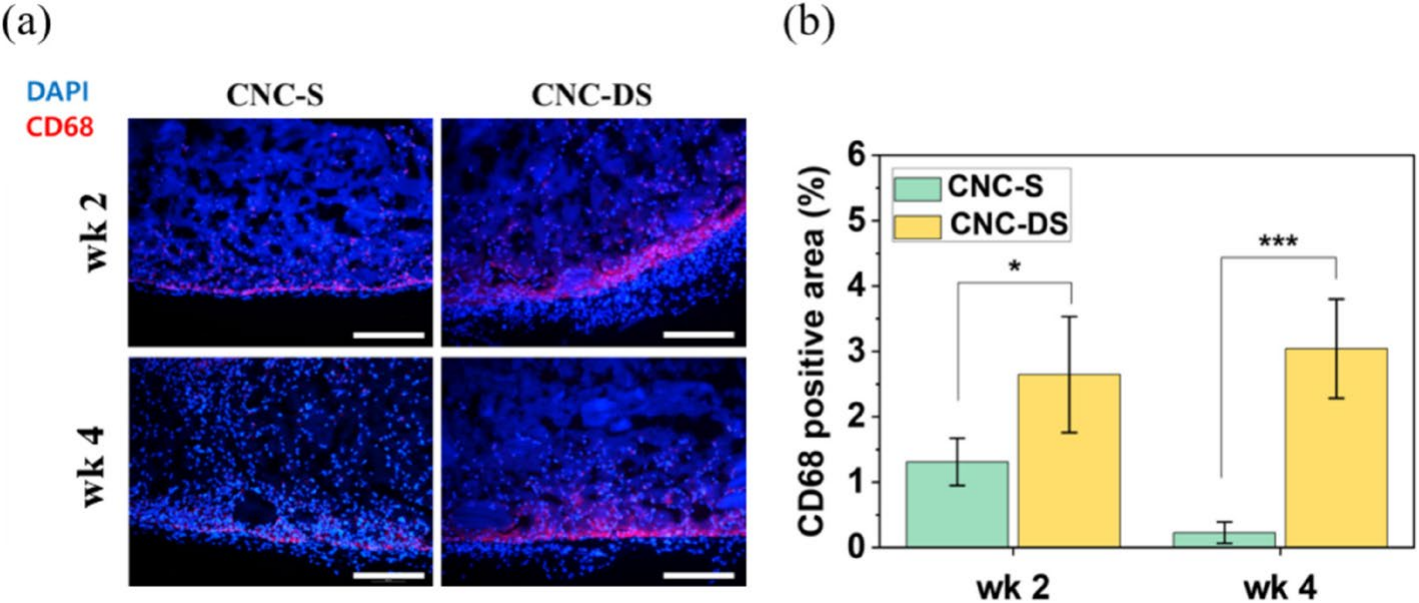
**a** CD68 immunofluorescence staining for subcutaneously injected CNC hydrogels at wk 2 and wk 4 (Scale bar = 200 μm). (blue: DAPI, red: CD68) (**b**) CD68 positive areas in CNC hydrogels (*n* = 5). (*: *p* < 0.05, ***: *p* < 0.001)

The immunofluorescence staining for macrophage (CD68) was carried out for the excised hydrogels to analyze the inflammation and foreign body response more specifically. Macrophages inside the CNC hydrogels were shown in red while counterstaining with DAPI was shown in blue in Fig. 5(a). The percentages of CD68 positive area in the images were measured and plotted in Fig. 5(b). Overall, a small number of macrophages were observed mainly on the edge parts of the hydrogels, where the collagen capsules were formed and the contact with skin and muscle tissues was made along with little macrophages infiltrated inside the hydrogel for CNC-S hydrogel at week 2. At week 4, macrophages were reduced significantly. This tendency was well-matched with the collagen capsule thickness data in Fig. 4(b) and (c). Also, by comparing macrophage signals and DAPI signals in Fig. 5(a) (as well as H&E staining data (Fig. 4(a)), the majority of infiltrated cells inside the hydrogels were not macrophages.

### In vivobioactivity of VEGF-loaded CNC hydrogel

**Fig. 6:**
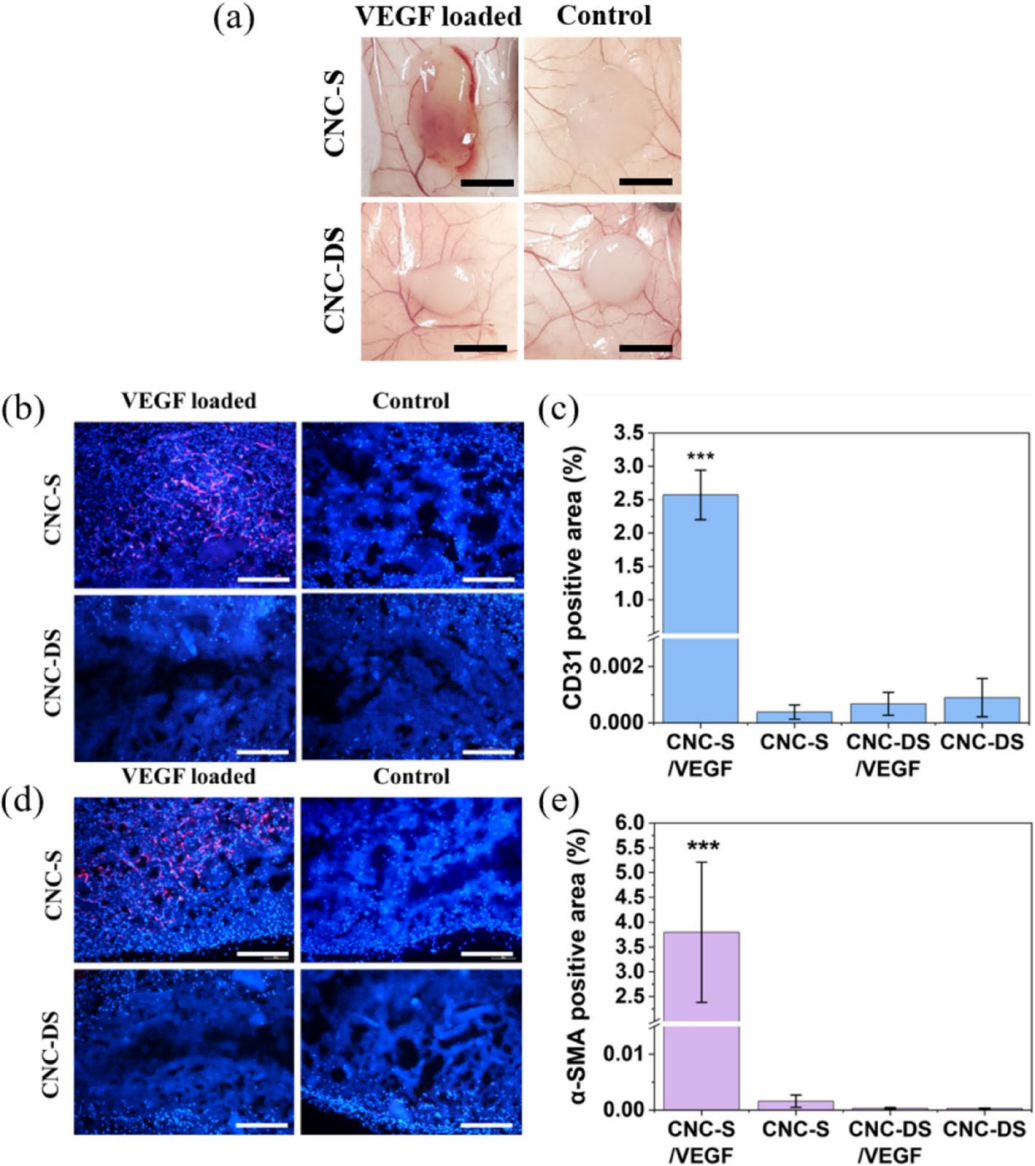
Angiogenesis induced by VEGF-loaded CNC hydrogels. **a** The images of CNC hydrogels attached on the skin tissues (Scale bar = 5 mm). **b**, **d** CD31 and α-SMA immunofluorescence staining images (blue : DAPI, red : CD31/α-SMA), and (**c**, **e**) CD31 and α-SMA positive areas (*n*=5) of CNC hydrogel sections at wk 2 (Scale bar = 200 μm). (***: *p*<0.001)

To endow a biological activity to CNC-S hydrogel, VEGF was loaded, and the induction of angiogenesis by VEGF-loaded CNC-S hydrogel (CNC-S/VEGF) upon subcutaneous injection was analyzed and compared with other groups, including VEGF-loaded CNC-DS (CNC-DS/VEGF), CNC-S, and CNC-DS without VEGF loading. After 2 weeks, the development of angiogenesis inside and around the injected hydrogel was clearly observed with the naked eye for CNC-S/VEGF whereas much less or no angiogenesis was observed in other groups, as shown in Fig. 6(a).

Unlike the previous Figs. 4 and 5 showing the edge parts of the gels, Fig. 6 represented the images of the middle part of the hydrogel. Cells (blue dots stained by DAPI) were abundant in the middle of CNC-S gels (both VEGF loaded/unloaded), whereas infiltration of cells was limited in CNC-DS hydrogel even with VEGF loading in Fig. 6(b) and (d). To characterize the infiltrated cells into the gels, the hydrogels were sliced and stained with blood vessel formation-related antibodies. CD31, also known as platelet endothelial cell adhesion molecule (PECAM-1), is one of the representative markers for endothelial cells, and α-SMA is the main component of the vessel wall and micro-vessels. CD31 positive staining images were shown in Fig. 6(b), and the percentages of the positive area were plotted in Fig. 6(c). CNC-S/VEGF showed dramatic blood vessel formation inside the hydrogels, while CNC-S without VEGF showed no blood vessel formation at all. Thus, the development of angiogenesis resulted not from the inflammation associated with the intrinsic CNC-S, but from the loaded VEGF inside the hydrogel. Interestingly, the noticeable blood vessel formation was not observed from CNC-DS/VEGF, as well as CNC-DS. α-SMA staining also showed a similar trend to CD31 staining overall (Fig. 6(d) and (e)). The expression of α-SMA was abundant in CNC-S/VEGF. In contrast, all other groups including CNC-DS/VEGF showed the rare expression of α-SMA.

## Discussion

This study aimed to evaluate the potential of CNC-S hydrogel as a scaffold for tissue engineering. CNC-DS was prepared as a negative control to analyze the effect of the sulfate group on the biological functionalities of CNC-based hydrogel. Minimal changes in physicochemical properties between CNC-S and CNC-DS hydrogels were desired to exclude the effect of other factors except for the sulfate group. Indeed, there were no noticeable changes in the morphology and size of CNC molecule after desulfation. Although aggregates of several nanocrystals were observed for CNC-DS compared to a single particle state for CNC-S in a dried state sample (Fig. 1(d)), the tendency of easier aggregation for desulfated CNC is presumably due to the reduced charge repulsion and increased hydrogen bonding among CNC molecules [40]. Regardless of desulfation, the addition of PBS to CNC- S or CNC-DS resulted in the stable gel formation, as previously reported that the gelation of CNC solution occurred by the addition of NaCl due to their intermolecular attractive interaction by charge screening [28, 29]. In the frequency sweep test, CNC-DS revealed a little lower G’, G”, and complex viscosity values than CNC-S for all frequencies, probably due to the higher aggregation tendency among CNC-DS after desulfation. Also, a little higher viscosity of CNC-DS observed at low shear rates (< 0.1 s^−1^) in Fig. 2(c) reflects the higher aggregation tendency of CNC-DS. However, CNC-DS hydrogel showed a bit lower viscosity at high shear rates (> 1 s^−1^), implying more severe destruction of the physical gel state of CNC-DS compared to CNC-S upon fast deformation in a non-linear regime. This may suggest a small but positive role of the sulfate group of CNC-S to form a physical, injectable gel state. Nonetheless, both CNC-S and CNC-DS hydrogels did not allow any degradation in a physiological buffer for 60 days. Cellulose (thus CNC) is known to be a biodegradable polymer, however, only by microorganisms, not by hydrolysis or in mammalian physiology [41].

Even though there was no significant difference in rheological properties and stability between CNC-S and CNC-DS, the release profile of VEGF was dramatically different from each other. Thus, the affinity of CNC itself to VEGF was poor, but the effect of the sulfate group of CNC-S on increasing the affinity to VEGF was sufficient enough to achieve the sustained release of loaded VEGF. Many growth factors and cytokines including VEGF are positive charges, so negatively charged CNC can bind with them electrostatically. Especially, the sulfate group was used as a key heparin-mimicking moiety that can contribute to providing binding affinity to various growth factors and cytokines [21, 22], and the same effect was observed for CNC-S. VEGF released from CNC-S showed very similar bioactivity to VEGF itself (pristine VEGF), demonstrating no bioactivity loss of VEGF at all. Thus, it could be concluded that the bioactivity of VEGF released from CNC hydrogels was well maintained.

As described in the Background section, the biocompatibility of CNC itself and CNC hydrogel in vivo has been controversial and not been clearly reported. In most cases, CNC was used as an additive to hydrogel composites to improve their mechanical properties or as a gelation inducer [24, 30, 31, 33, 42–44], and the reports for biomedical applications and biocompatibility investigations of CNC itself are limited [45]. In this study, hydrogels composed of only CNCs were subcutaneously injected into mice to evaluate their in vivo biocompatibility, which is the most basic characteristic of a biomaterial scaffold. When the body recognized injected or implanted hydrogels as foreign materials, a collagen-rich capsule would form to isolate them from the surrounding tissue [46]. Thus, thick fibrous capsule formation, implying not acceptable biocompatibility, would hinder the functions of implanted biomaterials [47]. Overall, the fibrous capsules formed around CNC hydrogels were not very thick for both CNC-S and CNC-DS cases; other biocompatible hydrogel systems in previous studies reported a similar degree of fibrous capsule thickness. For example, polyethylene glycol hydrogel induced ~ 50 μm of the fibrous capsule at day 28, and no significant difference was observed even after modification with zwitterion for reducing foreign body response [47]. Also, subcutaneously implanted poly(N-isopropyl acrylamide) (PNIPAAm)-based biosensor showed ~ 30 μm of the fibrous capsule after 30 days [48]. In addition, hyaluronic acid-based hydrogel formed ~ 30 μm of the collageneous capsule after 8 weeks [13]. Thus, both CNC-S hydrogel and CNS-DS hydrogel showed a low level of fibrous capsule formation, similar to other biocompatible hydrogels. Especially, CNC-S showed a much thinner fibrous capsule formation compared to CNC-DS, implying even milder foreign body response and better biocompatibility by the presence of the sulfate groups. In fact, sulfation has been utilized to enhance the biocompatibility of various polymers [49, 50]. Sulfated polymers showed enhanced swelling properties [51] and biocompatibility [52] as well as various biological activities. The reduced inflammation after 2 weeks was generally observed for other implanted hydrogels and materials [53, 54]. In the case of CNC-DS hydrogel, a significantly larger number of macrophages were observed compared to CNC-S hydrogel at the same time point whereas the location of macrophages and remaining of them over time were different from those of CNC-S hydrogel. So, the milder inflammation and better biocompatibility of CNC-S hydrogel compared to CNC-DS hydrogel were evident and significant, revealing the effect of the sulfate group on biocompatibility. Consequently, CNC-S hydrogel showed a much milder inflammatory response and foreign body response compared to CNC-DS hydrogel, while it also allowed the infiltration of cells inside the hydrogel, which is important and beneficial for biological activities, host integration, and tissue regeneration. Thus, the biocompatibility of CNC-S hydrogel was good enough for further biological applications in vivo. So far, peptide [6, 55], protein [4, 5] or ECM-based [11] scaffold materials have been reported to induce successful cellular infiltration. Our results demonstrate that CNC-S can be an emerging candidate that allows cellular infiltration inside the scaffold.

As mentioned in the Background section, the sulfated CNC can also mimic heparin, which is highly sulfated and has binding affinities to various growth factors as well as other biological activities [22, 49]. Many previous studies incorporated CNC into polysaccharide or collagen-based hydrogels for growth factor/cytokine delivery [30, 56–59]. However, similar to the studies of biocompatibility, most of these studies limited the role of CNC as an additive for the enhancement of mechanical properties. One study reported the effect of sulfation of incorporated CNC in a platelet lysate-based hydrogel on the modulation of stem cell response, based on different populations of adsorbed growth factors [60]. In our study, the biological role of sulfate groups of CNC as a growth factor binding site was investigated using CNC-only hydrogels.

Induced angiogenesis was observed after 2 weeks of VEGF-loaded hydrogel implantation subcutaneously. Both CD31 and α-SMA staining images showed evident and intense angiogenesis inside the hydrogel by VEGF loading only for CNC-S whereas almost no effect was observed for CNC-DS (Fig. 6). Thus, proper delivery (sustained release over several weeks) of VEGF was crucial for achieving the biological activity (angiogenesis) of the loaded growth factor. On the other hand, the results should not be overestimated that VEGF delivery by CNC-S could induce stable vessel formation in vivo. There have been many studies reporting that multiple growth factors (e.g., VEGF/PDGF) are necessary to induce mature vessel formation inside the matrix [61, 62].

In summary, CNC hydrogels had good biocompatibility in vivo with no or mild inflammation and foreign body response. Especially, CNC-S showed superior biocompatibility to CNC-DS and previously reported biomaterials. Also, the bioactivity of VEGF loaded in CNC-S hydrogel was preserved well, revealing efficient angiogenesis upon subcutaneous injection, while the bioactivity of VEGF loaded in CNC-DS was not verified in vivo. All of the results demonstrated the importance of the sulfate group on CNC for its biological activities. Furthermore, CNC hydrogels were favorable to cell infiltration from the outside, an important advantage for tissue engineering materials.

## Conclusions

CNC-S as an injectable hydrogel could also provide the sustained release of loaded VEGF while maintaining its bioactivity. Upon subcutaneous injection, CNC-S showed good biocompatibility and cellular infiltration owing to the physically associated structural micro-porosity and mild foreign body reaction. The loaded VEGF induced intensive angiogenesis development, showing strong expression of CD31 and α-SMA inside the hydrogel. In contrast, CNC-DS without sulfate group showed the burst release of VEGF, and it also induced more severe inflammation and fibrous capsule formation. CNC-DS induced almost no angiogenesis by VEGF loading. Since CNC-S has the potential to deliver not only VEGF but also other growth factors and cytokines due to its sulfate groups and allowed host cell infiltration and angiogenesis by VEGF loading, it has good potential for various biomedical applications including wound healing and tissue regeneration.

## Supplementary Information

**Additional file 1:** **Figure S1.** Size distribution of CNC-S and CNC-DS particles. **Figure S2.** SEM images of CNC hydrogels. **Figure S3.** Frequency sweep of CNC hydrogels dependent on the concentration of PBS. **Figure S4.** The complex viscosity of CNC hydrogels dependent on the concentration of PBS.

## Data Availability

The datasets used and/or analyzed during the current study are available from the corresponding author on reasonable request.

## Abbreviations

CNC: Cellulose nanocrystal
CNC-S: Sulfated cellulose nanocrystal
VEGF: Vascular endothelial growth factor
CNC-DS: Desulfated cellulose nanocrystal
PEG: Polyethylene glycol
bFGF: Basic fibroblast growth factor
EGF: Epidermal growth factor
HGF: Hepatocyte growth factor
BMP-2: Bone morphogenetic protein-2
CCK-8: Cell Counting Kit-8
DIW: Deionized water
FT-IR: Fourier-transform infrared spectrometer
XPS: X-ray photoelectron spectroscopy
EA: Elemental analyzer
DLS: Dynamic laser scattering
TEM: Transmission electron microscope
PBS: Phosphate-buffered saline
HUVEC: Human umbilical vein endothelial cell
ELISA: Enzyme-linked immunosorbent assay
CD68: Cluster of differentiation 68
CD31: Cluster of differentiation 31
αSMA: Alpha-smooth muscle actin
H&E: Hematoxylin and eosin
PECAM-1: Platelet endothelial cell adhesion molecule-1
PNIPAAm: Poly(N-isopropyl acrylamide)
