# Supplementary material for: Cellular infiltration in an injectable sulfated cellulose nanocrystal hydrogel and efficient angiogenesis by VEGF loading
Source: Biomater Res. 2023 Apr 10;27:28. doi: 10.1186/s40824-023-00373-y (PMC10084697; doi:10.1186/s40824-023-00373-y)
Supplement: Supplementary file 1 — Additional file 1: Figure S1. Size distribution of CNC-S and CNC-DS particles. Figure S2. SEM images of CNC hydrogels. Figure S3. Frequency sweep of CNC hydrogels dependent on the concentration of PBS. Figure S4. The complex viscosity of CNC hydrogels dependent on the concentration of PBS. [file 40824_2023_373_MOESM1_ESM.docx]

**Supplementary Information**

**Cellular infiltration in an injectable sulfated cellulose nanocrystal hydrogel and efficient angiogenesis by VEGF loading**

Kiyoon Min^a^ and Giyoong Tae^a.^*

^a^School of Materials Science and Engineering, Gwangju Institute of Science and Technology, 123 Cheomdan-gwagiro, Buk-gu, Gwangju 61005, Republic of Korea

* Corresponding author : [gytae@gist.ac.kr](mailto:gytae@gist.ac.kr) (G.Tae)

Tel.: +82 627152305


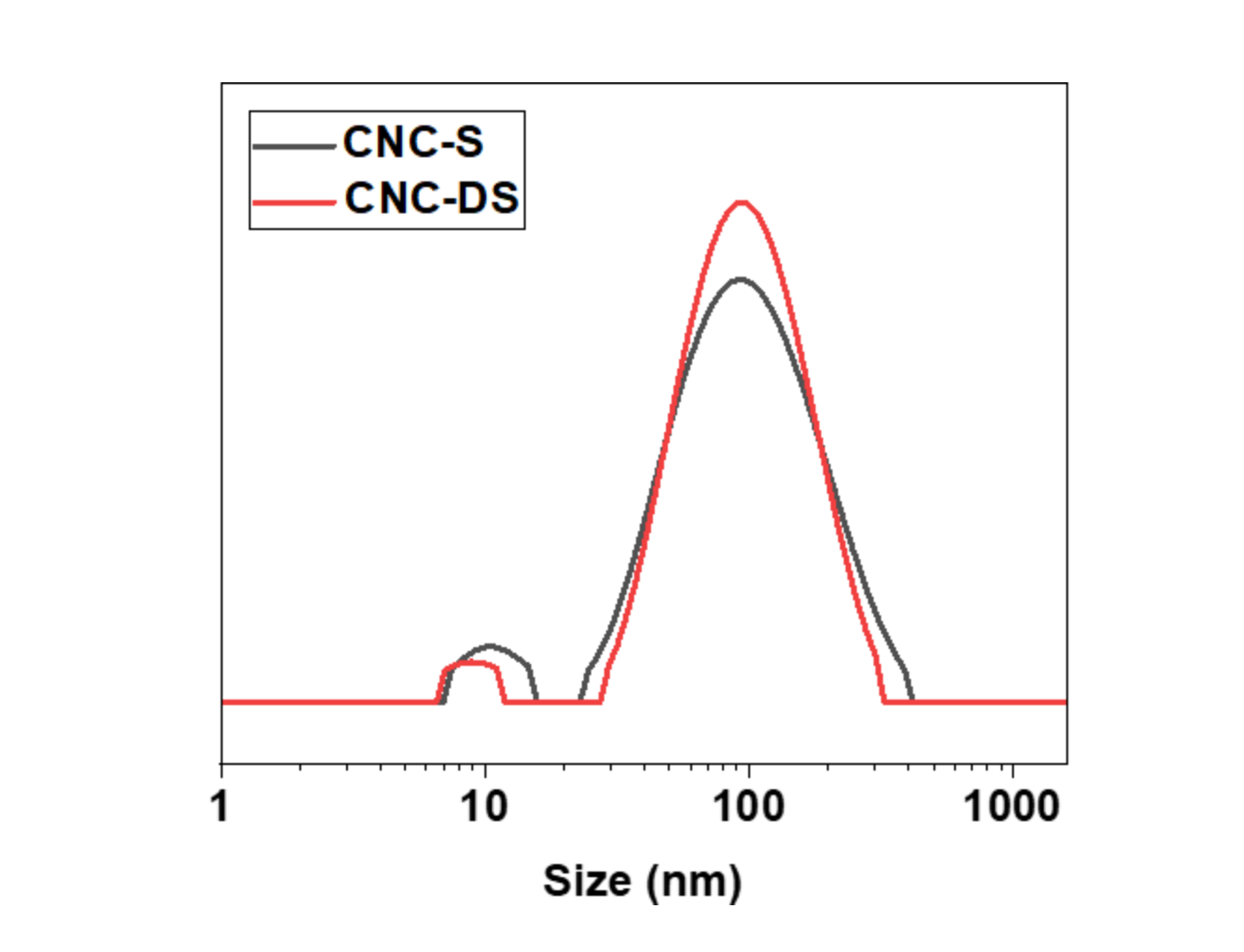


Fig S1. The size distribution of CNC-S and CNC-DS particles dispersed in DIW with Tween 80 (0.05%), measured by dynamic light scattering.


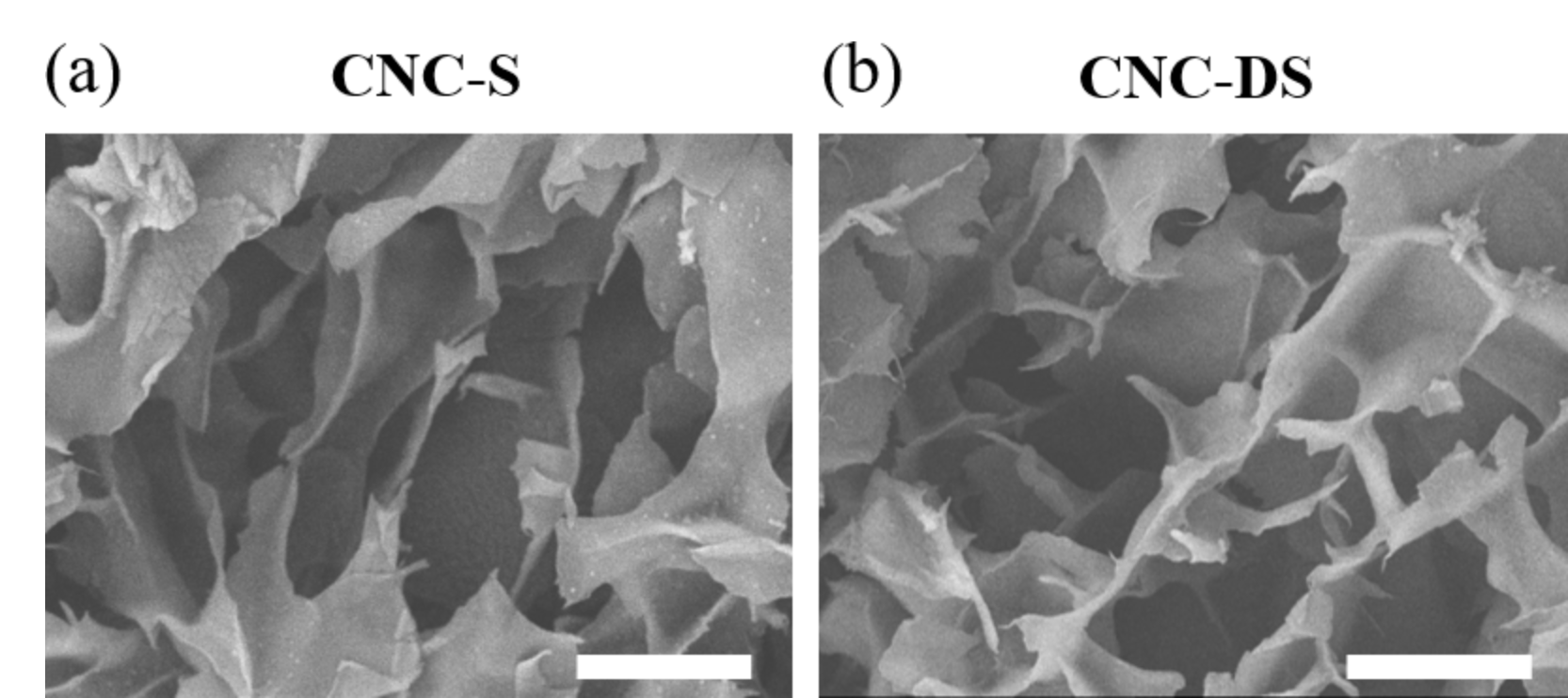


Fig S2. The morphologies of (a) CNC-S and (b) CNC-DS hydrogels observed by SEM (scale bar = 10 $\mu$m).


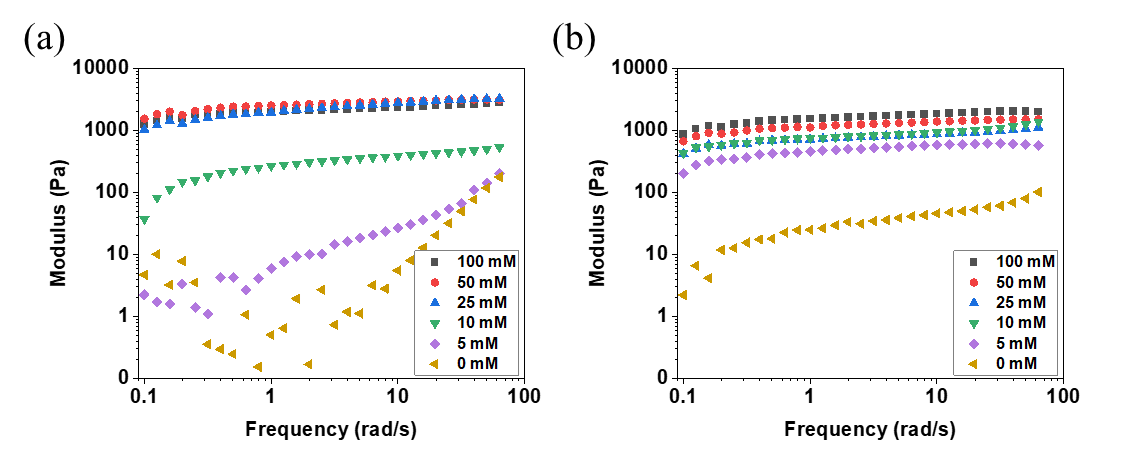


Fig S3. The frequency sweep measurements of (a) CNC-S and (b) CNC-DS hydrogels (4 wt%) in various NaCl concentrations of PBS.


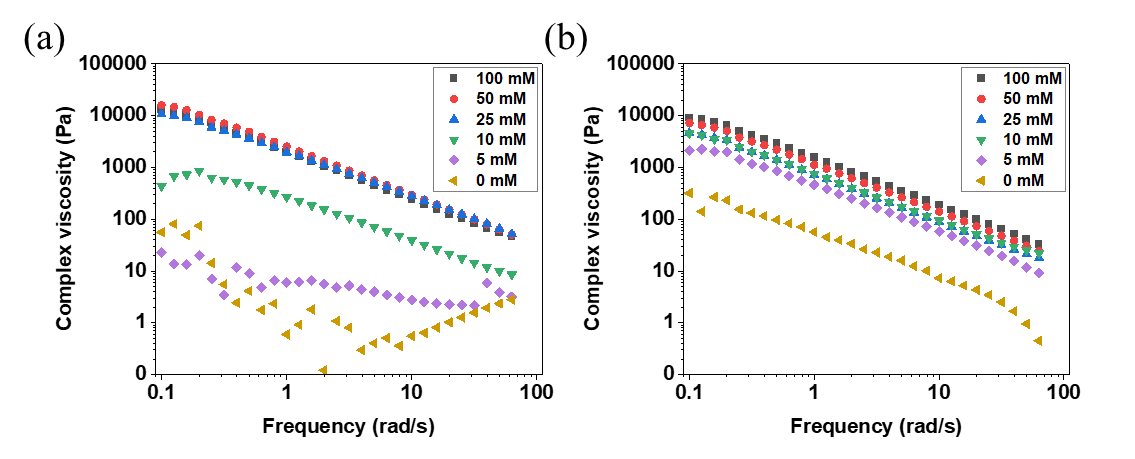


Fig S4. The complex viscosity of (a) CNC-S and (b) CNC-DS hydrogels (4 wt%) in various NaCl concentrations of PBS.
